# The Philosophy We Believe and the Principles We Practice

**DOI:** 10.4103/0974-777X.52974

**Published:** 2009

**Authors:** Editors, Publisher and Supporters of JGID

**Affiliations:** **Editorial Office:** Journal of Global Infectious Diseases, C/O: Dr Ricardo Izurieta, Department of Global Health MDC 56, College of Public Health, 12901 Bruce B Downs Blvd, Tampa, Florida, 33612, USA

The Board of Editors and the International Infectiologists Network in partnership with Medknow Publications proudly present the Journal of Global Infectious Diseases (JGID), which will promote the Translation of Science from Bedside to Benchside and Beyond in the field of Infectious Diseases across the world.

## THE SCOPE OF TRANSLATION SCIENCE

Today the time has come that Basic scientists need to work side by side with Clinical Researchers and public health experts to advance science which is helpful to the health of humanity. With this unique interaction between basic scientists, public health experts and clinicians the discoveries made in laboratories can be applied to human situations on a population basis. It is known that Investigations and interventions with human health are a result of scientific studies from genetics, cell biology and animal research. This knowledge is then translated to create diagnostic and therapeutic advances and innovations. The mission of translational research is to incorporate the discoveries from basic science areas into clinical investigations and public health studies and to use the results to generate research topics for basic science. JGID will promote such novel research.

Infectious Diseases research has its foundations in basic laboratory medicine. Translational Research is considered the pathway of application for ideas and innovations from basic science studies to the diagnosis, treatment and prevention of human disease. This area is the most dynamic and active scientific discipline. In 2005, “better translation of findings into patient care through guidelines” was at the top of the list of seven research priorities of the CDC, USA.

**Our Editorial Team** has been meticulously chosen from infectious experts around the globe and spans from areas of virology, bacteriology, mycology and vaccinology. Newer areas of nanotechnology, Computational Biology and Biological intelligence have also been incorporated.

**We will publish** scientific contributions such as Original Articles, Review Articles, Case Reports, Case Series, Clinical Trials, Clinical Investigations, Interesting Photographs, Radiology and EKGs and Expert Commentaries. The journal is peer reviewed and indexed. Article Submission and Peer Review is fully Online and Web Based.

**We believe** that Science will grow if shared and hence we are keeping JGID as open access. We also are going to have a SURGE Method which is a unique pathway to promote the publication of research via guidance by experts.

## SCIENTIFIC UPSCALING OF RESEARCH VIA GUIDANCE BY EXPERTS (SURGE)

SURGE is a Concept Founded and Promoted by the Journal of Emergencies, Trauma and Shock (JETS) and adopted by Journal of Global Infectious Diseases. (JGID)

Research is conducted to add knowledge on a topic. Research interests vary across the globe. If science has to grow it is important that research be accepted, inspected and respected from every corner of the world. There are many times that research material is good but lacks the presentation format which is needed to package and present the outcomes.

The mission of the JGID Team is to allow investigators across the world to have to their pleasure a high quality international publication to publish and promote research for others to gain knowledge.

We at JGID intend to maintain our high quality by accepting research of high quality. We also intend to help good research and researchers improvise their work to bring it to a super quality which meets global standards.

The SURGE Initiative is something which the JGID Team prides to be associated with. The way SURGE works is as soon as an article is submitted to JGID the Editorial Administrator decides about the potential of the article for publication. When the article is considered as a potential publication it is assigned to Expert Internal Editors who then decide whether it will go for External Peer Review or it will stay back for Expert Guidance for improvisation before being sent out for peer review.

The improvisation process involves personal guidance by Expert Internal Editors to the authors via emails. This process involves implementing suggestions regarding writing skills, grammatical correction, study design layouts, data analysis, presentation, and referencing and outcome modifications. The Experts make an extra effort to spend time on such articles so that the articles reach a quality high enough to meet the standard of JGID.

Scientific Writing is an art learnt over time. Convincing Editors to publish is like selling a product based on written material alone. Getting the message across in best possible manner with least possible hurdles is a skill which needs practice over and over again. Using correct sentences, words, phrases, messages are all a part of convincible research writing leading to acceptable publishing.

Today the international research arena is dominated by nations where research and researchers flourish. This has lead to disparity in research communication merely due to difference in standards of research across nations with economic variations.

We at JGID hope to bridge the gap as we believe in equality of research. If quality is a question we at JGID strive hard to make it standardized. SURGE allows scientists with good research but deficient publishing skills to better their work for the global audience at a level equally applicable to all across the globe.

**We invite** Clinicians, Nurses, Basic Scientists, Public Health Experts and Policy Makers to join our mission and use JGID as a vehicle to publish their research, commentaries, and experiences thus bringing the global scientific community together.

Please visit our website. www.jgid.org

**Continuous interaction and exchange of ideas will lead the progress of science.**

**Welcome aboard!**

### Further Information: Journal of Global Infectious Diseases (JGID)

JGID is an international peer-reviewed journal founded by a network of experts across the globe who recognized themselves as the International Infectiologists Network. The mission of JGID is to promote and publish infectious diseases research in areas of basic sciences, clinical medicine and public health.

JGID encourages research, education and dissemination of knowledge in the field of Infectious Diseases across the world thus promoting translational research by striking a synergy between basic science, clinical medicine and public health. The Journal intends to bring together scientists and academicians in Infectious Diseases to promote translational synergy between Laboratory Science, Clinical Medicine and Public Health. The Journal invites Original Articles, Clinical Investigations, Epidemiological Analysis, Data Protocols, Case Reports, Clinical Photographs, review articles and special commentaries. Students, Residents, Academicians, Public Health experts and scientists are all encouraged to be a part of this initiative by contributing, reviewing and promoting scientific works and science

The journal will be published semiannually in 2009, four monthly in 2010 and quarterly from 2011. (March, June, September and December).

The journal allows free access (Open Access) to its contents and permits authors to self-archive final accepted version of the articles on any OAI-compliant institutional/subject-based repository. The journal does not charge for submission, processing or publication of manuscripts and even for color reproduction of photographs.

**Online Submission Site:** www.journalonweb.com/jgid

### Bibliographic listings for JGID:

The journal is indexed with Caspur, DOAJ, EBSCO Publishing's Electronic Databases, Expanded Academic ASAP, Genamics JournalSeek, Google Scholar, Health & Wellness Research Center, Health Reference Center Academic, Hinari, Index Copernicus, OpenJGate, SCOLOAR, Ulrich's International Periodical Directory

The journal will be published semiannually in 2009, four monthly in 2010 and quarterly from 2011. (March, June, September and December).

### Medknow Publications

Medknow Publications, a publisher of open access peer-reviewed, scientific journals, is committed to the improving the visibility and accessibility of the science from the developing world. Its endeavor in continuously re-inventing the publishing methodology for about a decade has resulted in high quality peer-reviewed scholarly journals. Medknow pioneers in ‘fee-less-free’ model of open access publishing and provides immediate free access to the electronic editions of the journals without charging the author or author's institution for submission, processing or publication of the articles.

Medknow, with over 80 print + online journals, is probably the largest open access publisher of print journals in the world which does not charge author or author institution for submission, processing or publication of articles. Each journal published by Medknow has its independent website. The websites use the OpenURL standard, making it easy for libraries to link users as directly as possible from citation to the full text of the article. These journals are searchable from DOAJ (www.doaj.org) and are part of HINARI. All the journals provide RSS feeds which is also ATOM enabled. All the issues are also available in XML format. The open access policy has resulted in more than a half a million article downloads in a month for all the journals.

